# Research and application of polypropylene: a review

**DOI:** 10.1186/s11671-023-03952-z

**Published:** 2024-01-02

**Authors:** Md. Tanvir Hossain, Md. Abdus Shahid, Nadim Mahmud, Ahasan Habib, Md. Masud Rana, Shadman Ahmed Khan, Md. Delwar Hossain

**Affiliations:** 1 Department of Textile Engineering, Bangladesh University of Business and Technology (BUBT), Dhaka, 1216 Bangladesh; 2 Department of Textile Engineering, Dhaka University of Engineering and Technology, Gazipur, 1707 Bangladesh

**Keywords:** Polypropylene (PP), Polymer, Nanomaterial, Membrane, Applications

## Abstract

Polypropylene (PP) is a versatile polymer with numerous applications that has undergone substantial changes in recent years, focusing on the demand for next-generation polymers. This article provides a comprehensive review of recent research in PP and its advanced functional applications. The chronological development and fundamentals of PP are mentioned. Notably, the incorporation of nanomaterial like graphene, MXene, nano-clay, borophane, silver nanoparticles, etc., with PP for advanced applications has been tabulated with their key features and challenges. The article also conducts a detailed analysis of advancements and research gaps within three key forms of PP: fiber, membrane, and matrix. The versatile applications of PP across sectors like biomedical, automotive, aerospace, and air/water filtration are highlighted. However, challenges such as limited UV resistance, bonding issues, and flammability are noted. The study emphasizes the promising potential of PP while addressing unresolved concerns, with the goal of guiding future research and promoting innovation in polymer applications.

**Graphical Abstract:**

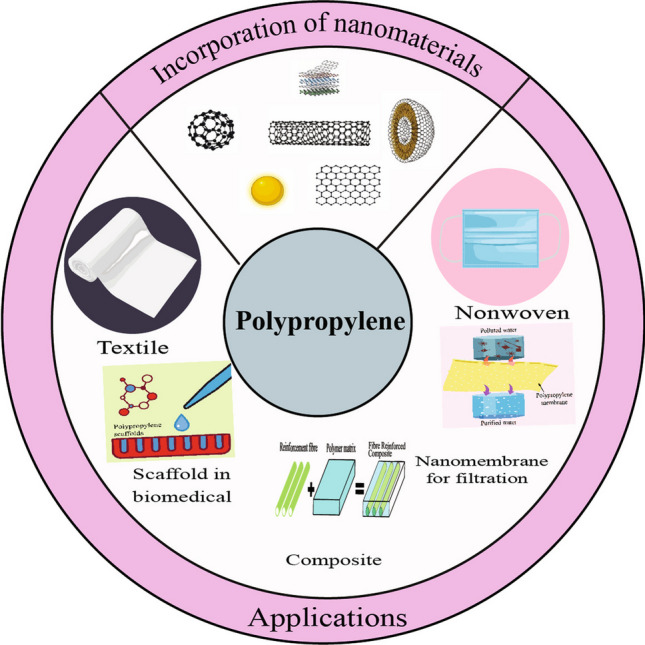

## Introduction

In recent decades, PP has emerged as one of the most widely used thermoplastic polymers due to its exceptional properties, cost-effectiveness, and ease of processing [1]. Over the years, significant progress has been made in developing and utilizing PP, leading to its widespread applications across various industries [2]. This article aims to highlight a comprehensive overview of the most recent advancements in PP research and its versatile applications while exploring the future outlook for it [3].

The versatility of PP originates from its unique molecular arrangement, which consists of propylene monomers joined together in an unbent chain. This linear configuration proposes several benefits, including increased crystallinity, superior chemical resistance, inferior density, and satisfactory mechanical strength [4]. These features make PP eligible for various applications, from packaging materials and customer products to automotive elements and medical devices [5].

In current years, investigators have focused on improving the properties of PP through various techniques such as copolymerization, modification with additives, and nanocomposite formation [4, 6]. For instance, Green et al. revealed insulation of PP and propylene-ethylene by using a copolymerization approach [7]. Mastalygina and his colleagues improved the properties of PP by modifying it with low-density PP and powdered cellulose [8]. W Liu et al. reported an overview of the electrical properties of PP-based nanocomposite [9]. The improved thermal stability, improved impact resistance, outstanding barrier properties, and enriched electrical conductivity have been observed by using the above-mentioned techniques.

PP is a flexible material with a wide range of uses. It enhances filtration and antifouling properties in membrane technology. PP composites reinforced with carbon nanotube (CNT) or jute fibers improve mechanical strength and electromagnetic shielding. Scaffolds made of PP have potential for use in biomedical applications [10]. PP is frequently used in textiles, and it strengthens concrete. PP is applicable across industries because of its adaptability and versatility [11].

Applications have been categorized in the form of PP, including membrane, composite, and fiber. They will encompass a broad spectrum of applications of PP, including but not limited to water filtration, air filtration, biomedical, packaging, apparel, automotive, aerospace, construction, and recycled materials [12, 13]. Each application category will be discussed in detail, highlighting the key advancements, challenges, and potential future directions.

The incorporation of nanomaterial like graphene, MXene, nano-clay, borophane, silver nanoparticles, etc. paid a good attention over the years. Gawish et al. [1] produced a PP hybrid composite with graphene nanoplates in the yield. The tensile strength showed a greater change of approximately 16 MPa to 33 MPa, while the flexural strength and impact energy enhanced by 16% and 12%, respectively. To produce medical orthopedic devices with PP and CaCO_3_ nanoparticles that could be used instead of natural bone, and it could also be used as a thermal insulation material [14]. Many such researches have reported in this article.

Moreover, the applications of PP have increased over the decade; however, a comprehensive review of the applications of PP has been found limited. Maddah reported a PP review focusing on plastic materials [15]. Himma et al. reported preparation, modification, and applications as only membrane form [16]. Siracusa focused on bio-based polymers, including PP [17]. Chung and his team’s review aims only at energy storage applications [18].

Thus, this review article aims to provide a thorough overview of the most recent developments in PP research and their numerous applications. It will be invaluable for researchers, engineers, and business professionals working with PP because it examines recent developments, difficulties, potential outcomes and new ideas. This article is anticipated to encourage additional innovation, advance sustainable utilization, and support the continued success of PP across a range of industrial sectors.

## Fundamental and chronological development of PP

**Fig. 1:**
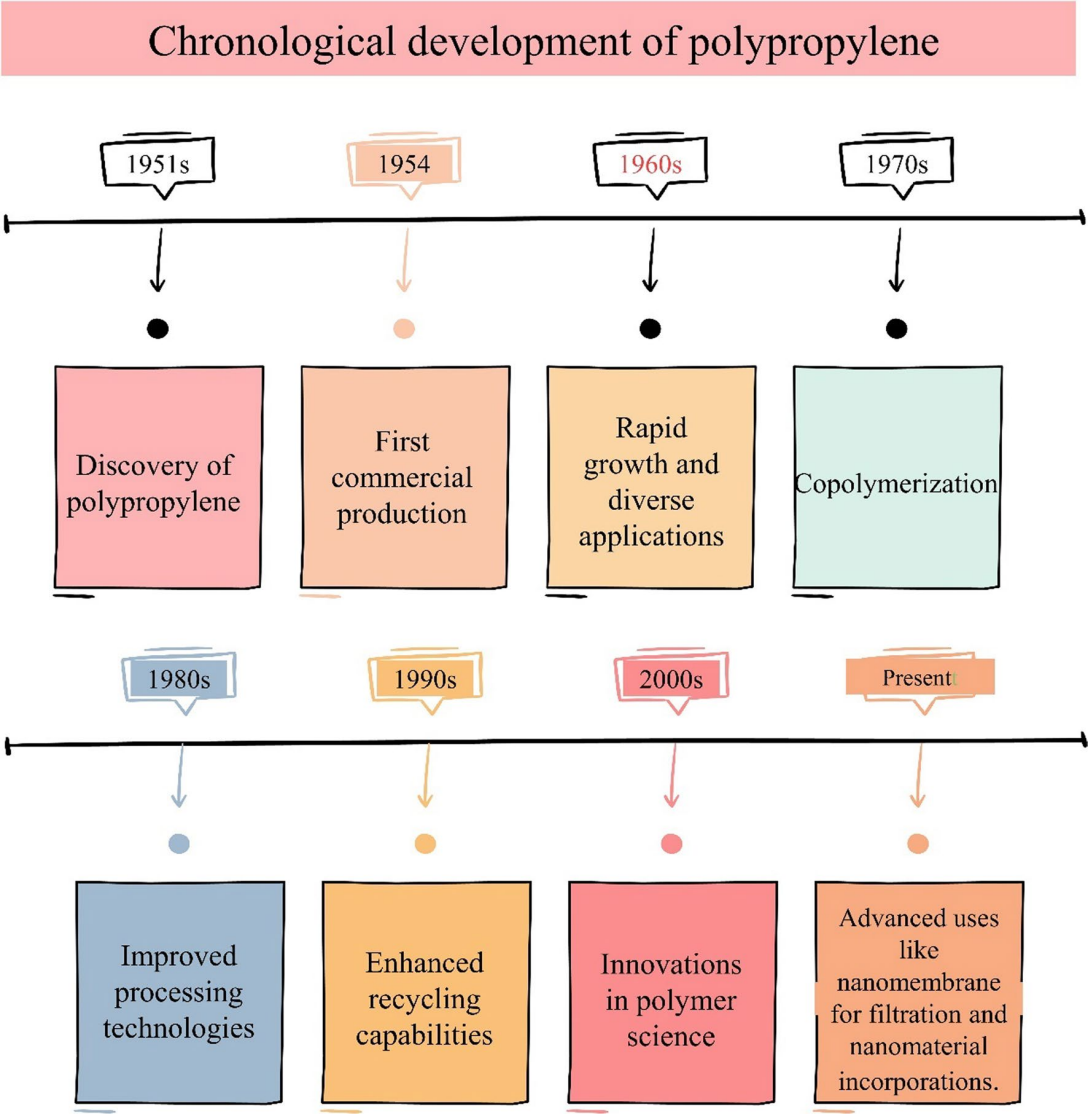
Chronological development of the PP over the decades

PP is a robust, stiff, and crystalline thermoplastic. It is generated from the monomer propene (or propylene). PP was discovered by scientists Paul Hogan and Robert Banks by accident while working at the Phillips Petroleum Company in 1951. After that, Phillips Petroleum Company began the first commercial PP manufacturing in 1954. The material was first sold as PP, then as "Marlex. In 1957, the stereospecific catalysts were introduced by Karl Ziegler and Giulio Natta's research results in creating stereospecific catalysts, allowing the synthesis of isotactic PP with better characteristics. Then, PP acquired popularity in the 1960s because of its outstanding mechanical and chemical qualities. Its use is quickly expanding in various sectors, including packaging, textiles, automotive, and consumer products. After that, the development of copolymerization processes enables the production of random copolymers and block copolymers of PP, allowing for more control over characteristics and application-specific tailoring in the 1970s. Improvements in processing methods have been observed since 1980s, such as injection moulding, extrusion, and blow moulding, making PP more versatile, allowing for the manufacture of complicated forms and structures. Then, when recycling capacities increased in the 1990s, PP's recyclability became a critical attribute in its widespread use. Recycled PP maintains its properties, making it a viable option for ecologically responsible applications. Polymer science advancements in the 2000s and continuous polymer science research and innovation resulted in the invention of new grades and formulations of PP, increasing its range of characteristics and applications. Finally, at present days, the PP is one of the most often used polymers on a worldwide scale. Its adaptability and ongoing innovation keep it at the forefront of contemporary production and everyday living. PP has witnessed constant developments and broad acceptance throughout its history, making it a standard in various sectors owing to its cost-effectiveness, adaptability, and excellent performance in a wide range of applications. Chronological development of PP is shown in Fig. 1.

## Advancement of incorporation of nanomaterial with PP

PP has versatile uses in nanotechnology, and it is engaging in new fields day by day. PP could be used as a home appliance to spacecraft to create a better future and a more sustainable world. Researchers have been given greater attention over the last few decades and are trying to improve the performance of PP by mixing or blending with other nanomaterials.

To know the different properties of PP, some recent researches are analyzed below the table, and their preparation methods, application, characterization, processing challenges, as well as future aspects are also discussed. The Table 1 clearly shows that PPs are used with different nanoparticles, such as Gawish et al. [1] produced a PP hybrid composite with graphene nanoplates in the yield. The tensile strength showed a greater change of approximately 16 MPa to 33 MPa, while the flexural strength and impact energy enhanced by 16% and 12%, respectively. Since conventional PP has transformed into a hybrid composite, the application sector has changed to high-impact consumption. To produce medical orthopedic devices with PP and CaCO_3_ nanoparticles that could be used instead of natural bone, and it could also be used as a thermal insulation material [14]. Y. Shi et al. produce wearable electronics and energy storage devices, Mxene nanoparticles are used with PP, which is a revolutionary change for the modern biomedical sector as well as smart textile production [19]. This MXene-base composite material incredibly increases tensile modulus and ductility. H. Palza as wearing clothes for humans, antimicrobial properties are essential. Copper nanoparticle (NPCu)-based PP-coated textiles showed higher antimicrobial properties; only 5% volume of PP/NPCu can remove 99.8% of *S. aureus* germs within 60 min [20].

**Table 1:** Summary of the method, characteristics, and application of the nano-particle integrated PP material

Entry	Nanomaterial	Method	Characteristics	Application	Challenge	References
1	Graphene nanoplatelet	Injection moulding	Tensile strength: enhanced from 16 ± 1.5 MPa to 33 ± 2.1 MPa; flexural strength: increased from 50.7 MPa to 58.8 MPa; impact strength: improve 132%	Solar panel components, piping systems, bicycle frames, helmets, etc	Nanomaterial’s alignment is challenging	[85]
2	Silver nano	Coating	Antimicrobial:0.44 to 2.60% increased	Medical and hygiene	Difficult to control parameter during silver nano particle extraction of AgNO_3_ and NaOH	[86]
3	Iron nano	Melt interaction	Tensile strength: 24% increased; air permeability: 33% reduced	Food packaging	Non-oxidizing environment must need to prepare nano particle	[83]
4	Organoclay	Melt mixing	Tensile strength: enhance (15%-17%); tensile modulus: increase (61% -133%); interlaminar fracture toughness improve 67%	Rotor blades, building panels, structural components, electronic housings	Large-scale applications can be challenging, recycling very difficult	[60]
5	Nanocellulose	Melt extruder	Tensile strength improve from 30.4 MPa to 36 MPa	Biodegradable packaging materials. biocompatible medical devices	Improvement of flexibility	[87]
6	Graphene nano	Melt blending	Tensile strength: enhance up 34% thermal stability: increase from 431 $^\circ{\rm C}$ to 473.5 $^\circ{\rm C}$	Automotive components, antistatic packaging	Temperature controlling difficult because of two different materials	[88]
7	Silica nanoparticles	Immersing	Thermal stability: increased up to 937 mAh/g	Lithium-sulfur batteries	PP immerge into hydrolysis solution	[89]
8	Nano-CaCO_3_	Injection moulding	Tensile strength improved 17.9MPAa -19.3 MPa; flexural strength increased up to 50%	Medical orthopedic device, thermal insulation, porous filtration	Controlling the size and distribution of cells in the foam, interactions between nanoparticles and the polymer matrix	[14]
9	Oil palm nano filler	Hand lay-up	In adding 3% nano filler. tensile and impact strength: increased 60.8% and 27.6%; adding 6% filler, tensile and impact strength increased 56% and 29%	Structural components, device housings, wind turbine blades, panels and structural elements	The production and handling of nano fillers may raise health and safety concerns	[21]
10	Graphene Nanoplatelet	Melt mixing	Thermal stability: 1% GNP adding thermal stability increase up to 50%	Heat sinks, battery components, barrier films	Recyclability,	[90]
11	Graphene nanoplatelets	Surface coating	Tensile strength: 1 wt% xGnP is used strength increased 13.6% but 3 wt% xGnP showed 8% decrease simultaneously tensile modulus increased 41.7% at 3 wt% xGnP	Automobiles industry, aerospace, marine, and other industrial applications	Interfacial bonding	[91]
12	Nano clays	Melt blending	Tensile strength: above 5% wt adding decreased strength, tensile modulus is increased	Casing and housings, children's toys, durable industrial components, panels	Homogeneous dispersion of nano filler, chemical compatibility between filler and polymer matrix	[92]
13	MXene Nanosheets	Melt blending	Tensile strength enhance 35.3%; tensile modulus: increase 102.2%; ductility: increase 674.6%	Wearable electronics, thermal management materials, energy storage devices	Dispersion of MXene nanosheet, achieving and maintaining the electrical challenging	[19]
14	Ti_3_C_2_T_x_ MXene	Vacuum compression molding	Only 2.12 vol % of MXene showed conductivity 437.5 Sm^−1^	Conductive films and coatings, environmental sensors	Interfacial bonding, processing conditions, electrical conductivity	[93]
15	Copper oxides nanoparticles	Coating	Antimicrobial property: PP Cu^2+^ showed 10% better than PP Cu^+^	Textile fabric, footwear, medical bandage	Long-term stability, durability and wash ability of incorporated nanoparticles	[94]
16	Copper nanoparticles	Coating	Antimicrobial test: at 5% volume of PP/NPcu, *S. aureus* reduce up to 99.8% within 60 min	Water treatment	Fastness	[95]

N. Saba Oil palm nanofiller PP hybrid composites are versatile structural components; they could be used as wind turbine blades, car panels, and different types of complex structural designs [21]. The interesting matter is that with only 3% of oil palm nanofiller, tensile strength increased by 60.8% and impact strength was enhanced by 27.6%. To produce different types of PPs nanoproducts, researchers faced lots of challenges, including the incorporation of nanoparticles, different parameter control, durability, large-scale production, and recycling processes. In spite of so many challenges, there is a lot of possibility for future work on PP, such as borophene nanomaterial, which could be used to produce high-load bearing materials. It could be used with graphene and carbon-base advanced materials. Since PP is a non-biodegradable material, it could react with biodegradable co-polymerization to be used as a biological transplanting and self-healing material.

## Advanced functional application areas

There are numerous cutting-edge functional applications for PP in multiple industries. It is suited for a wide range of specialized applications thanks to its exceptional combination of qualities, including excellent chemical resistance, low density, outstanding fatigue resistance, and superior electrical insulation [22]. Table 2 summarizes the features, applications, and fabrication of advanced PP-based membranes.

**Table 2:** Summary of PP-based membrane fabrication, feature, and application

Sl. No	Materials	Fabrication technique	Feature	Application	References
1	PP, zeolite, polycarbonate	Melt blown	Highly efficient and can filter PM_2.5_	Air filtration	[96]
2	PP, zeolitic imidazolate, copper(II), benzene-1,3,5-tricarboxylate	In-situ growth of nano-metal–organic frameworks	Ultra-stable PP membrane	Air filtration	[97]
3	PP	Electrospinning	Nanoparticle filtration by symmetric PP hollow-fiber membranes	Air filtration	[98]
4	PP, CNT	Melt Electrospinning	Higher conductivity, high tensile strength, excellent hydrophobicity	Filters, protein adsorption and desorption, electrochemical sensors, tissue engineering, protective clothing	[99]
5	Syndiotactic PP	Electrospinning	High AC breakdown strength and high melting point	High voltage cable insulation	[100]
6	PP, polyvinyl alcohol	Solution electrospun and melt electrospun combined	Reject more than 96% of the 0.5 µm particles	Water filter	[101]
7	PP, poly (vinylidene fluoride), sodium perchlorate	Electrospinning	High ionic conductivity, high discharge capacities, excellent cycle performance	Battery	[102]
8	PP, polyvinyl alcohol	Electrospinning	More than 99% salt rejection	Water filter distillation	[103]
9	β-nucleated isotactic PP, β-nucleating agent (WBG)	Electrospinning	Tensile strength increases with increasing the WBG	Filtration, protective clothing, composites reinforcement, tissue engineering	[104]
10	PP, polyvinyl alcohol, zeolite midazole frameworks-8	Melt-blown electrospun	Filtration efficacy 96.5% for PM_2.5_, 33.34N tensile strength	Air filtration	[105]
11	PP, poly (ethylene glycol)	Solution electrospinning	Rapid catalytic reduction of methylene blue dye	Water filter	[106]
12	PP	Electrospinning	Water removal from ultra-low sulfur diesel efficiencies reaching 99%	Water filter	[107]
13	PP	Melt-electrospinning	Protection performance 95%; air permeability 100 cm^3^/s/cm^2^	Air filtration, water filter	[72]
14	PP, polyhedral oligomeric silsesquioxane-chloropropy, poly(vinylidene fluoride)	Electrospinning	Porosity 63.0%, electrolyte uptake 271.4%; tensile strength 31.6 MPa; elongation at break 39.0%,	Battery	[108]

## PP membrane

PP is one of the most popular polymers for making membranes, which has excellent thermal stability, chemical resistance, mechanical strength, and affordability. PP electrospun membrane is widely used in water filtration, air filtration, biomedical fields and many more due to its micro-sized and nano-sized fiber, high specific surface, and ease of spinning [23]. Numerous studies have documented the use of PP membranes in water filtration. However, developmentsthis fieldsection this section will highlight the recent development in the scientific arena regarding these fields.

### Water filter

**Fig. 2:**
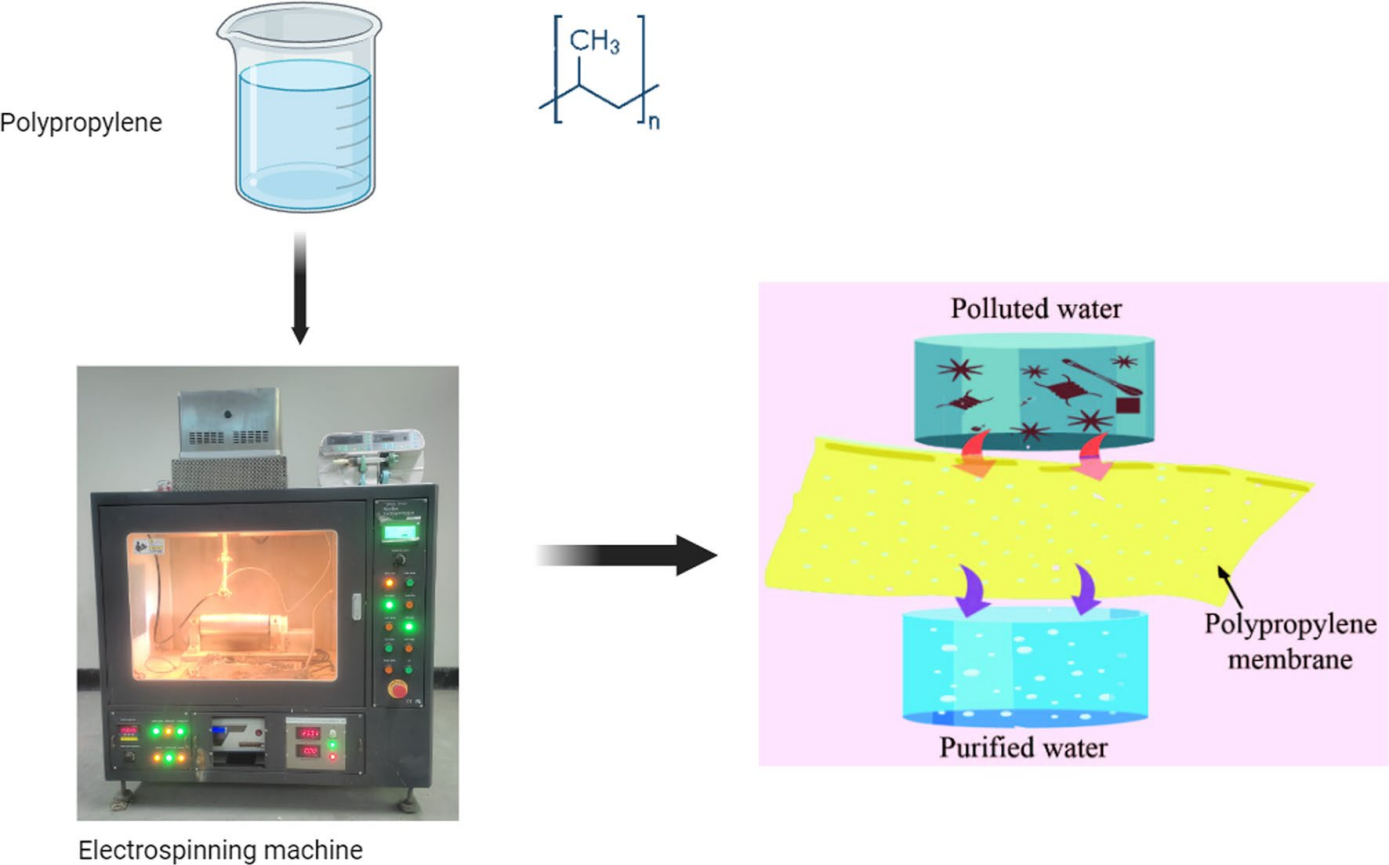
Schematic diagram of the PP-based water filtration mechanism

PP-based filter membrane for water treatment, especially waste water; sea water desalination and so on, has gained much attention over the years. A schematic diagram of the PP-based water filtration mechanism is shown in Fig. 2.

Electrospinning technology is widely used to prepare the filtration nano-membrane. A based nano filter membrane for the treatment of waste water derived from textiles was disclosed by Zakaria and colleagues. They have created nanofibers with 228 nm on average in diameter. By contrasting their filter with a commercial PP membrane that has more mechanical strength, they indicated that their filter is promising [27]. However, limitations have been observed in terms of degradation in contact with chemicals (strong acid, base, organic solvents). Another article was published where they claim that developed membrane is capable of removal of microplastics from urban waste water [24]. However, susceptibility to fouling is still a limitation that must be overcome.

Besides, oil-form-water separation is emerging significant for its economic value, and many research articles have been published in recent years [25–28]. For example, PP based nano-membrane for oil–water mixture separation is reported by Wang et al. [29]. Sea water desalination has paid interest for the water crisis over the years. Promising research is going on to advance this steam. Marek Gryta applied plasma treated PP-based nano-membrane for desalination [30]. In another study, the PP/chitosan membrane is prepared and characterized [31]. Similarly, many studies carried out regarding the PP-based membrane for removing salt from seawater [32–34]. Future scope available to work on the membrane’s filtering rate incrassation, improving the quality of the membrane, reducing cost, and so on.

### Air filter

**Fig. 3:**
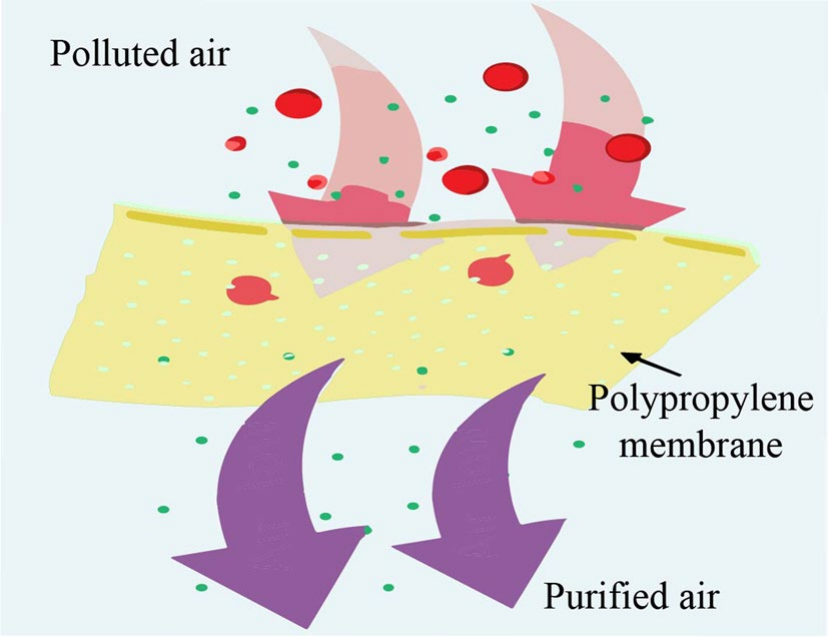
Schematic diagram of the PP-based air filtration mechanism

Various polymers have been used for air filtration over the years [35]. PP and other polymers is surging massive attention in many diversified applications, including air filtrations, as illustrated in Fig. 3. Many research works are progressing on this topic around the world. For instance, Deng and colleagues reported Janus microsphere filter media, which omit the existing limitations of commercial facemasks like limited moisture, vapor permeability, and lower microorganism inhibition [36]. In addition, metal nanoparticle incorporated PP membrane shows 91.68% efficiency for air filtering by Cheng et al. et al. [37]. Oher’s techniques, including melt blowing, are also used to prepare PP membranes. For example, Deng et al. prepared a PP-based air filter, which shows 99.87% filtration efficiency. Getting homogeneous fiber structure is still challenging, and we should focus on decreasing the standard deviation to prepare fiber diameter [38]. Therefore, the researcher has the potential to enhance this field of study.

### Biomedical field

The use of PP polymer in the biomedical fields is less widespread than PVA and others owing to its non-biocompatibility and non-biodegradability features [39]. However, the addition chitosan, collagen, and other medicinal substance-based mat has become popular over the years. Ahmet and team studied PP/chitosan-based mat as a scaffold the area for biomedicals [40]. Ganjalinia et al. reported PP with a Lactic acid-based mat for use as a scaffold in the body [41]. Also, a mesh with PP, after modifying its surface, has been prepared for the preparation of biomedical devices [42]. PP/CNT and hydroxyapatite nanocomposite in human subchondral bone are produced by Khan et al. [43].

**Fig. 4:**
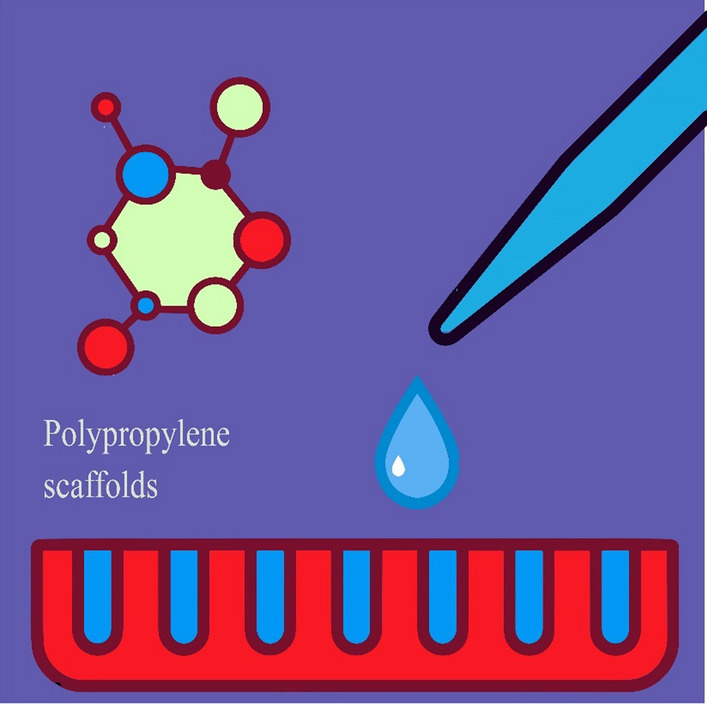
Schematic illustration of the PP-based scaffold for biomedical application

PP/cellulosic fiber-based bio-composite has been prepared for artificial organs and tissue engineering. They have not done any in vivo or in-vitro analysis, just reported mechanical properties. Experimental results do not fully support that their bio-nano-composite is promising for biomedical applications [44]. Table 3 provides an overview of PP-based materials, including fabrication techniques, characteristics, and notable applications and Fig. 4 shows a schematic illustration of the PP-based scaffolds for biomedical application.

**Table 3:** An overview of PP-based materials including fabrication methods, characteristics, and noteworthy applications

Entry	Form of PP	Materials used	Fabrication technique	Property	Application	References
1	Mesh	PP, stabilizer	Polymer extrusion	Use for hernia repair	Biomedical	[109]
2	Scaffold	PP, chitosan, hydrogel, TiO_2_	Electrospinning	Useable as a antibacterial scaffold	Biomedical	[110]
3	Fiber	PP fiber	Spinning, weaving, sewing	Finer cloth for sportswear, cold clothing gear	Cloth	[111]
4	Fiber	Sand, cement, PP, and others	Concrete preparation	Suitability to use in concrete to improve the mechanical performance	Construction	[112]
5	Scaffold	PP polymer and tricalcium phosphate (TCP) ceramic	Fused deposition	Porous scaffold for bone grafts	Biomedical	[113]
6	Fiber	Short carbon fiber/PP with exfoliated graphene nanoplatelets	Melt blending and hot-press	Better tensile strength and impact properties	Automobile and constructions	[91]
7	Scaffold	PP fumarate, hydroxyapatite (HA) nanoparticles	Extrusion-based printing technique	Suitable for bone implantation	Medical science	[114]
8	Composite	PP, henequen fiber	Moulding	Higher tensile strength	Construction	[115]
9	Composite	Alkylated graphene oxide, PP	Bimolecular nucleophilic substitution reaction	Higher tensile strength	Construction	[116]
10	Nano Composite	Trimethoxy silanes, PP	Solution mixing	Better thermal stability, crystallinity and mechanical properties	Automobile	[117]
11	Composite	CNT, glass fiber and PP	Extrusion and injection moulding	Better tensile strength and modulus value than individuals	Replacement of glass or CNT in different purposes	[118]
12	Composite	Hexagonal boron nitride (*h*BN) with 1-dodecanol	Solution mixing process and reaction	Exhibit better thermal and mechanical properties with very low filler content	Interior in house	[119]
13	Nano composite	Titanium carbide (Ti_3_C_2_T_x_)/PP	Solution casting and melt blending	Mention initial degradation temperature (79.1 °C increase), tensile strength (35.3% increase), ductility (674.6% increase) and storage modulus (102.2% increase)	Used as thermally stable polymer in different purpose	[19]
14	Nano composite	Titanium dioxide (TiO_2_) nanospheres with pimelic acid and Iso tactic PP	Solution mixing and chemical reaction	Improved thermal stability and viscoelastic properties were found	Can be used as elastic	[120]
15	Composite	100% recycled cotton fibers and PE/PP	Hot press process	It showed sound transmission up to 8 dB	Can be used in automobile and interior design	[121]
16	Composite	PP and PE	Flat hot compact	High stiffness, low level of damping	Interior design	[122]
17	Composite	PP and graphene	“In-situ building” approach	Excellent heat dissipation	High power and highly integrated electronic devices	[123]
18	Composite	Graphite, PP	Solution mixing process and reaction	Excellent thermal conductivity	Electronic device	[124]
19	Scaffold	PP carbonate, PDLA and Beta TCP	Salt-leaching method	Better result for bone defect repairment	Biomedical	[125]
20	Scaffold	Dicalcium phosphate dihydrate, PP fumarate	Solution mixing process	Better bone tissue engineering	Biomedical	[126]
21	Composite	Lignin-based hollow carbon fibers, lignin/PP	Electro spinning	High performance to low weight ratio	Aerospace	[127]
22	Composite	PP, carbon powder	Extrusion and film blowing	Extremely low aqua absorption level with high impact resistance	Interior	[128]
23	Composite	PP, carbon fiber	Extrusion and film blowing	High strength and stiffness along with good temperature resistance to 215°F	Interior and construction	[129]
24	Composite	PP, cementitious	Solution mixing	Better flow strength, less displacement to flow and better rheological parameters	Construction	[130]

## PP composite

**Fig. 5:**
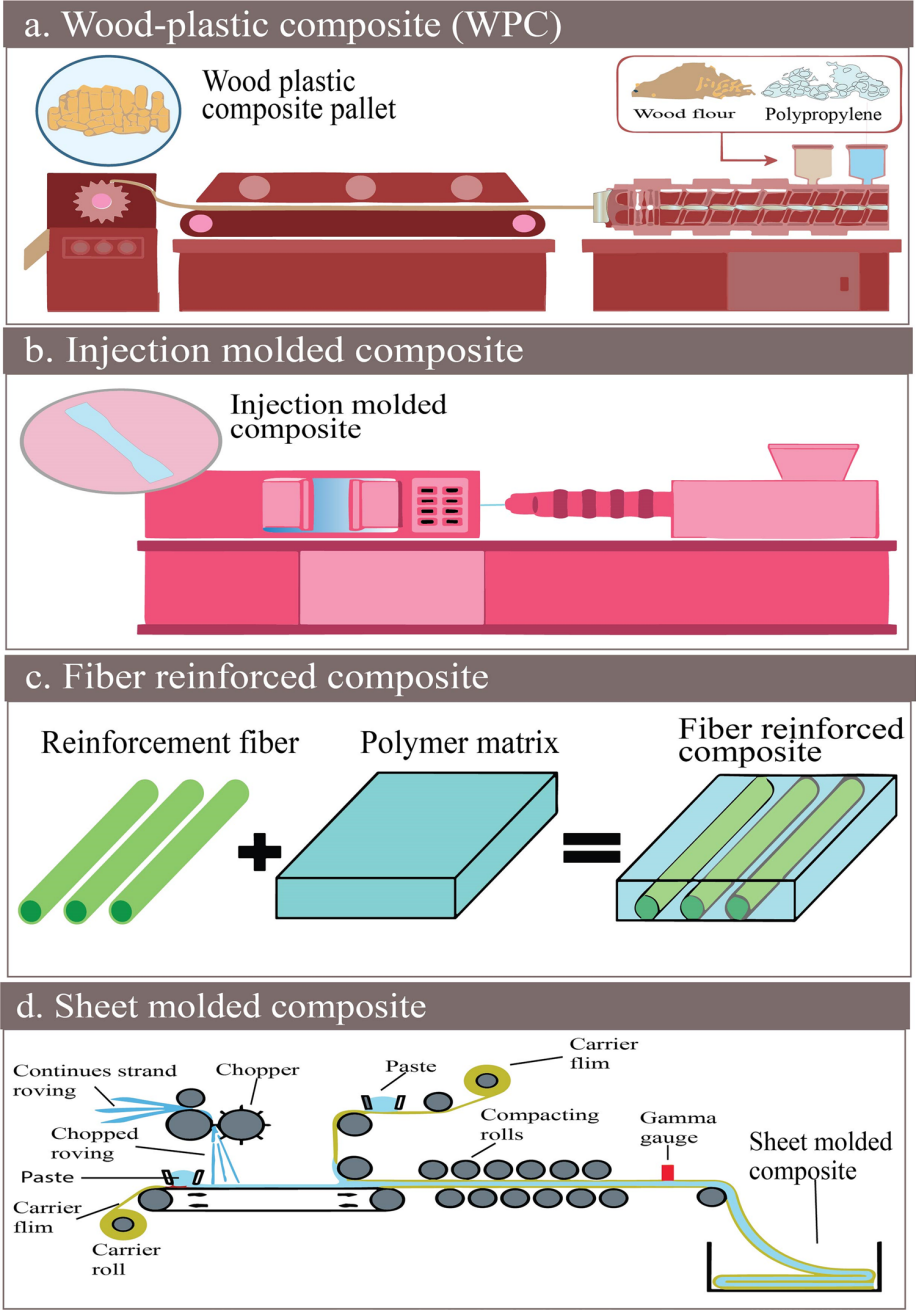
Preparation techniques of **a** wood plastics composite, **b** injection molded composite, **c** fiber reinforced composite, **d** sheet molded composite

The use of PP in the composite as is performing a significant role in the thermoplastic type composite. Different manmade, natural and high-performance fiber has been used as reinforcement over the century [45]. The preparation techniques for wood plastic composite (WPC), injection-molded composite, fiber-reinforced composite, and sheet molded composite are shown in Fig. 5a–d, respectively.

PP is frequently used as a matrix material in composite structures, where it functions as the continuous phase that binds the reinforcing material together. The following are essential applications of PP as a matrix in composite materials:

### Fiber-reinforced composite

PP is commonly used as the matrix material in fiber-reinforced composite, blending with carbon, glass, or natural fibers. Due to their low weight, high strength, and corrosion resistance, these composites are utilized in numerous industries, including the automotive, aerospace, and construction industries. Shen et al. investigated the jute fiber-reinforced PP composite to determined mechanical and acoustic properties. The mechanical properties improved by up to 50% of the fiber content of jute afterwards decreased [46]. On the other hand, the best acoustic insolation performance is found in the 50% fiber content. Anandjiwala and his colleagues study about flax fiber reinforce PP composite and its chemical modification [47]. The composites were made with zein-treated nonwovens flax fiber. These composites were tested for tensile, flexural, impact, and reinforcing characteristics. Chemically treated flax fibers increased mechanical parts in composites. Maurya et al. examine a sisal fiber base PP hybrid composite which produced by chemically activated fly ash. The results found that tensile and flexural properties increased 30.54% and 48%, respectively and the maximum impact strength was found to be 0.80 kJ/m^2^ [48].

### Wood-plastic composite (WPC)

PP is used as the matrix in wood-plastic composite when combined with wood or natural fibers. WPC have the aesthetic appeal of wood in addition to moisture resistance and low maintenance, making them ideal for outdoor decking, fencing, and furniture [49].

Jubinville et al. developed a highly loaded PP based WPC; it was found that PP may be recycled six times without losing most of its material qualities. It also found that chain scission caused by thermo-mechanical processing dominated PP alterations after re-processing. The high viscosity of virgin PP limited WF loading to 60 wt.%, and recycled PP allowed up to 70 wt.% WF loading [50]. Lin et al. examine how the three components of biomass and plastics interact during the pyrolysis of the WPC. Compared to their estimated yields, the experimentally obtained char yields with C-PP and H-PP rose, but the woody residue yield from L-PP barely reduced. The global output of WPC has grown and is still rising [51]. Kuka et al. examine the environmental characteristics of WPC made of PP and heat-treated wood. The results showed excellent performance of composite against UV light [49]

### Thermoplastic composite

PP is frequently used as a matrix material in thermoplastic composite, in which it may be repeatedly melted and reformed. These composites are utilized in vehicle components, consumer goods, and industrial equipment due to their ease of production and recyclability. Sultana et al. investigated the theoretical stiffness of short jute reinforcement PP thermoplastic composite. In the results short fiber preform composite provide extreme stiffness [52]. Arju et al. developed different woven and knitted structural jute fabric base PP thermoplastic composite and the result was found that the twill structure height value of tensile strength (48 MP) at twill structure was 134% higher than other plain fabric structures [53]. Kaewkuk and his coworker analyzes sisal fiber/PP thermoplastic composite where cellulose decomposition increases due to an increase of PP temperature. When fiber content was increased then decrease the pollution of cellulose [54]. In addition, PP is using as insulation cables and has good features in the industrial scale. For instance, Adnan and his team reported PP based nanocomposite for the use in insulation cables [55].

### Injection moulded composite

PP is frequently blended with short fibers or fillers in injection-moulded composite products to enhance its mechanical properties. PP composites are introduced and formed into home appliances, electrical enclosures, and car interior parts. Panthapulakkal & Sain developed a short hemp/glass reinforced hybrid PP composite in the inject moulding; it found that the mechanical properties of the hybrid composite increased with increased glass fiber content, and it was determined that 15% wt.% of short glass fiber content maximum amount of flexural strength and modulus [56]. Billah et al. examined continuous and discontinuous rattan fiber reinforced PP injected moulded composite where the tensile properties of discontinuous fiber enhanced up to 30%. On the other hand, the tensile properties of continuous fiber enhanced up to 59%[57]. Nuez et al. developed flax shives reinforced inject moulded PP composite, whereas the tensile strength and Young modulus tremendously increased due to good bonding with fiber and PP matrix [58].

### Sheet moulding composite (SMC)

As the matrix material, PP is utilized in the fabrication of SMCs, which are utilized to produce large, complex objects with high strength-to-weight ratios. SMCs are used for vehicle body panels, truck parts, and infrastructure components. Chatterjee et al. investigated the thermal behavior and mechanical properties of jute fiber PP composite; it was founded a strong bond between jute fiber and PP. Jute fibers and PP in the composites exhibited high tensile strength increased by 10% at 2-ply fiber loading. Due to polymer degradation caused by repeated processing, the mechanical properties of four-ply composites are diminished [59]. Gabr et al. found that the thermal and mechanical improved using a carbon fiber/PP composite by sheet moulding process [60].

### Thermoset composite

When combined with thermosetting resins such as polyester or epoxy, PP can function as a matrix material in thermoset composites. These composites in aerospace, maritime, and high-performance applications feature exceptional mechanical properties. Y. Li et al. study of carbon/PP and epoxy/PP thermoset composites revealed that the PP/epoxy and PP/epoxy/CB composites significantly reduced chain folding energy while increasing the rate of crystallization of the composites [61]. Abd El-baky et al. developed a PP glass thermoset composite to analyze the flexural strength and probability of failure of hybrid composites. In the results, the strength is increased by the hybridization of glass fiber with PP, and the static as well as flexural properties of the PFRP, GFRP, and P-G composites with hybridization are the following: 82.37, 88.4, and roughly 84.9%[62]. M. Li et al. produced a PP base thermoset composite and successfully improved mechanical properties because of the crosslink structure of epoxy resin [63]. PP has extensive use in the medical field for various applications like medical devices, diagnostic equipment, surgical instruments, and packaging and so on,due to its versatility, cost-effectiveness, and compliance with regulatory requirements in the healthcare industry [64].

## PP fiber

**Fig. 6:**
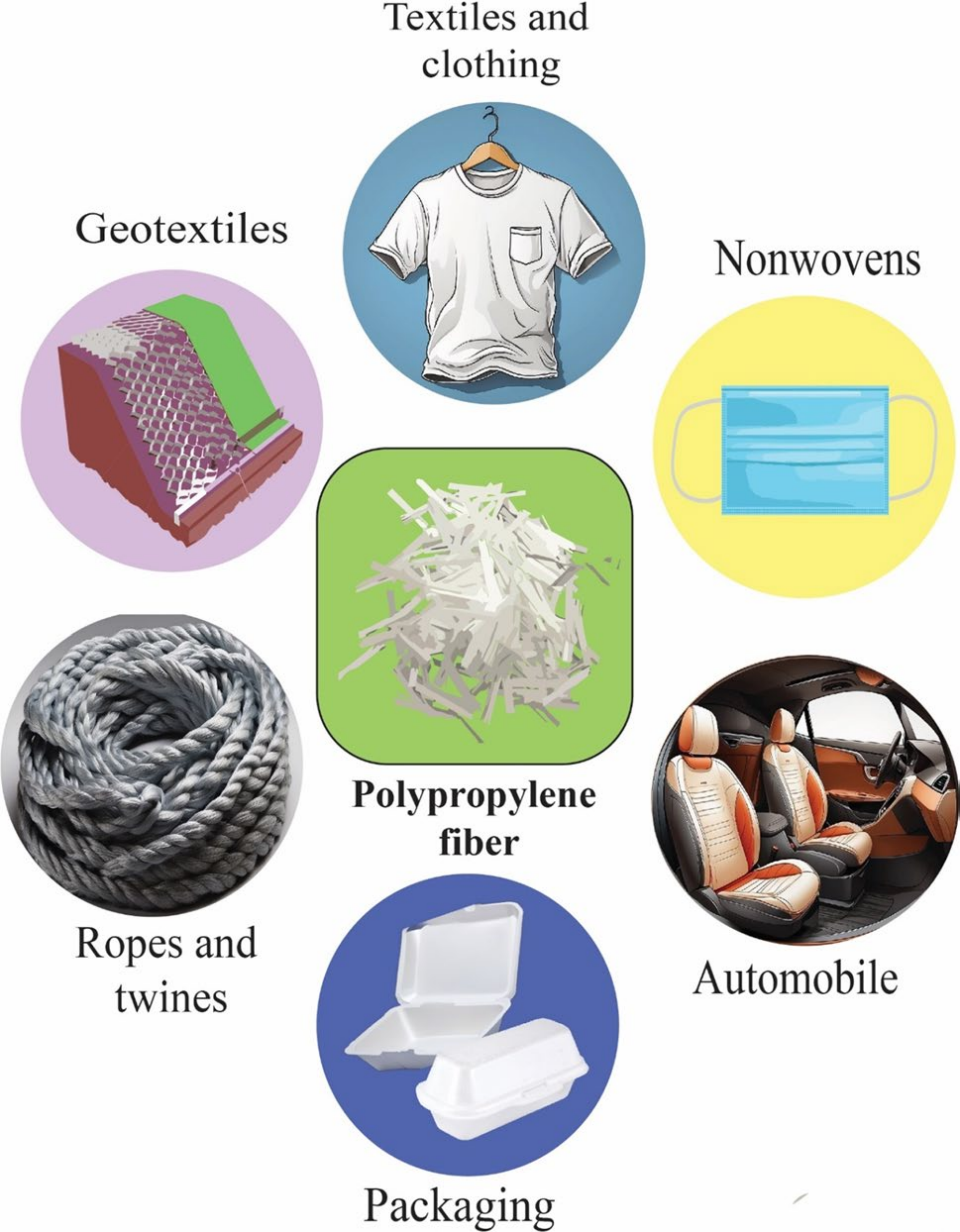
Schematic illustration of various applications for PP fibers

In fiber form, the PP is used in apparel (sportswear, cold clothing gear, underwear’s and so on), garments accessories, diaper top cover, non-woven facemask, PPE, and many diverse applications [65]. Also, PP fiber-reinforced concrete composites paid attention enormous have great attention, and huge scholarly articles have been published over the decade. For instance, concrete with PP fiber reinforcement and its use in the design of public interiors has been reported by Blazy et al. [66]. Yoan et al. report on reinforced concrete's mechanical characteristics and microstructure with glass and PP fibers [67]. Investigation of the mechanical and durability characteristics of concrete reinforced with glass and PP fibers have reported by Liu et al. [68]. On the other hand, PP fiber is also used for composite preparation. PP fiber reinforced composite with organic materials like gypsum prepared by Nguyen et al. [69]. Admittedly, Fig. 6 represents the various application of the PP in the form of fiber. Due to their advantageous qualities, PP fibers have a wide range of uses in several sectors. Among the most critical applications for PP fibers are textiles and clothing, geotextiles; automobiles, nonwoven; ropes and twines.

### Textiles and clothing

In the textile industry, PP fibers are widely used to manufacture a variety of fabrics and garments [65]. Due to their lightweight, suppleness, and capacity to wick away moisture, these fibers are ideal for athletic wear, activewear, and undergarments.

Uddin et al. analyzed personal protective clothing that was made from PP. this PPE was used worldwide to protect humans from Covid-19 [70]. In addition, Shahid et al. reported that PCM incorporated fabric for medical textiles applications [71]. Lee & Obendorf created a liquid protective textile utilizing electrospun PP web; this (EPWs) and laminates showed a combination of solid protection efficacy and an acceptable amount of air/vapor transport qualities [72]. Wang et al. investigated the possibility of using remaining PP fabric from carpet backing as edge trim to keep a polyethylene (PE) matrix. A single piece of used PP fabric that had been cleaned was placed between four layers of 0.1 mm thick PE film. Following that, they were moulded at a temperature of 150 °C and a pressure of 290 kPa. Comparing the resultant PE/PP composite's tensile strength and flexural modulus to those of pure PE, it was discovered that the composite, which included a 25% PP volume percentage, had increased three times and 60%[73].

### Geotextile

As geotextiles, PP fibers are widely used in civil engineering and building applications. As seen in the Fig. 6, PP geotextiles offer soil stabilization, erosion management, and drainage solutions. They are utilized in the construction of roadways, berms, and landfills. Valentin et al. investigations to pinpoint potential durability issues resulting from UV light exposure to geotextiles, using both macro- and micro-scale methods. The tested commercial PP geotextile deteriorated after 500 and 1000 h of UV exposure. The tensile tests revealed that following exposure to UV light, the material stiffness increased, along with the tensile strength and ultimate load elongation [74]. Rawal & Saraswat's research on needle-punched nonwoven geotextiles was made using different weight ratios of PP/viscose and polyester/viscose fibers. In the newly developed nonwoven geotextiles, the porosity decreases were calculated at predetermined atmospheric pressures of 2, 20, and 200 kPa. The 20/80 volume fraction of PET/viscose and 20/80 volume fraction of PP/ viscose materials showed the least amount of porosity decrease. However, at high normal pressures (200 kPa), PP/viscose and PET/viscose with 400 g/m^2^ of mass per unit area have produced the least porosity reduction [75].

### Automobile sector

In the automobile business, PP fibers are utilized for automotive carpets, seat covers, and interior trims. PP fibers are good for automotive interiors due to their durability, abrasion resistance, and ease of upkeep. Agarwal et al. analysis about ecofriendly lightweight PP polymer composite automobile used for racing car, window, board panel door opener because of low cost and reduced automobile weight [76]. Lyu & Choi analysis of the different types of bio composite showing PP base composites are highly used in the automobile industry due to their low cost, good chemical resistance and rapid solidity behaviors. In addition, this composite is frequently used in the door trim, bumpers, side panel, crash panel, wheel housing, guide channels and other parts [77]. Akampumuza et al. reported in this study due to environmental awareness most of the automotive industry is applying bio composites. To produce lightweight automotive parts, PP base bio composite were used since fuel consumption is directly related to the weight of the automobile. This study also reveals that a 25/15 weight fraction of hemp and glass PP reinforced composite provided extreme mechanical and thermal properties comparatively hemp reinforced composites; it could apply for better structural and high stiffness and thermal insulation [78].

### Nonwoven

Nonwoven fabrics used in various applications, including hygiene products (diapers, sanitary napkins), medical textiles (surgical gowns, masks), and filtering media, are frequently fabricated using PP fibers. Lou et al. studied recycled PP and polyester nonwoven selvages to make Acoustic composites with excellent noise absorption performance. High-frequency sound waves, most notably over 2000 Hz, were absorbed well by permeable composites. Enhancing composite depth improves middle and small frequency absorption of sounds. It decreases when composite density increases [79]. Kumeeva & Prorokova investigate whether Fluorinating nonwoven PP improves sorption. Oxygen-fluorination effectively improves material characteristics. Oxygen-containing molecules make the nonwoven a little hydrophobic. This material is promising for sanitary product manufacture since its water sorption capacity improves by 3–4. Due to the creation of low-energy fluorinated molecules on the PP fiber's surface as well as an increased the degree of roughness volatile fluorine and nitrogen processing boosts wasted oil capacity for absorption. Hydrophobic nonwoven PP polymers may be better for disposable health care cloth than typical materials [80].

### Ropes and twines

Ropes and cords are manufactured from PP fibers for a variety of industrial and commercial applications. PP ropes are desirable due to their high ratio of strength-to-weight, UV resistance, and buoyancy. Morais and his team reported the mechanical properties of PET and PP-based rope for multifunctional applications [81]. Also, Foster and his colleagues studied the fiber rope’s advantages over the wire-based ropes [82].

### Packaging

Packaging products such as woven sacks, bulk bags, and flexible intermediate bulk containers incorporate PP fibers. PP is suitable for heavy-duty packaging applications because of its high tensile strength and resistance to ripping. Allahvaisi and his team summarized the prospect of the PP polymer use in food packaging applications [82]. Also, Khalaj et al. reported the PP was modified with nano-clay for the packing applications. It is found to be a great prospect and has world has abundant market for this product worldwide [83]. Moreover, Phase change material integrated PP fabric can be use as food packing by using molding machine [84].

## Challenges and future scope

- Despite many benefits, PP with natural and synthetic polymers-based composite has some challenges, including high manufacturing time, long preparation time, low demand, sensitivity to moisture contact and many more. Attention should be paid to overcoming these challenges.
- PP-based electrospun mat is promising for filtration applications due to its structure, mechanical properties, and chemical properties. Biomedical, has many limitations, and the main one is lack of biocompatibility and biodegradability. To improve this collagen, chitosan, other medicinal using is observed. So, colossal research scope is available to work with those limitations and advance this field.
- In solution electrospinning, a complex getting effective solvent for the PP is difficult and hazardous for the environment. On the other hand, due to its thermoplastic properties, it is suitable for melt electrospinning. Still, it consumes a huge amount of energy and a long process in the heating chamber may cause the degrade polymer. Therefore, prospective researchers can find the research gap from this.
- PP fiber has been commercially used for a long time and applies in various apparel, home furnishings, and accessories despite its weak in UV radiation, poor bonding, high flammability, and so on. Therefore, attention should be given to improving UV degradation, flammability, bonding characteristics, and other features.
- Nano-materials integrated PP have huge prospects in the next generation nano-based products along with some obvious challenges like low production rate of electrospinning machines, occasional bead formations, limitations use of polymer, and many more. Future research can be directed to overcome those challenges.

## Conclusion

This comprehensive research has examined PP's most recent advances, incorporations with nanomaterials, and advanced applications. Since PP has versatile uses in nanotechnology, it is engaging in new fields day by day. It could be used as a home appliance to spacecraft to create a better future. Researchers have been given greater attention over the last few decades and are trying to improve the performance of PP by mixing or blending with other nanomaterials. There are several limitations on utilization in the domains of aerospace, automobiles, filtrations, and other applications that have been mentioned by carefully evaluating various fabrication techniques. In addition, promising research is going on to overcome those limitations and advance this field. Also, there is a research gap in working on it. The summarized latest applications in a table and mechanisms presented through figures would be easy for prospective readers to understand. There have been solutions for issues such as lengthy manufacturing time, lengthy preparation times, low demand, susceptibility to moisture contact, and many others. The future research roadmap is presented at the end of this paper. Moreover, it is crucial to work on the existing limitations or challenges of PP-based products to meet the customers’ demands. It is expected that this review can use a credible source for the researcher and industrialist.

## Data Availability

On reasonable request, the corresponding author will provide the datasets collected during and/or analyzed during the current work.
